# Cases in Obstetrical Practice

**Published:** 1860-12

**Authors:** B. Woodward

**Affiliations:** Galesburg, Ill.


### CASES IN OBSTETRICAL PRACTICE.

BY B. WOODWARD, M. D., GALESBURG, ILL.

### *Placenta Prævia and Post Partem Hemorrhage.*

Case 1st.—Sept 23rd, 1860.—This morning, as Mrs.———, near the close of her second pregnancy, was walking across her room, she was taken with a very slight pain, and a gush of blood per vaginum. Thought she had lost more than a pint of blood. Complained of pain in her head—she was not flooding when I found her—pulse 110; os not dilated. Ordered her to take 4 drops of verat. vir. every three hours till the pulse came down, which it did to 70, soon after taking the third dose; and to keep the recumbent posture. Through the day there was very slight oozing of blood, but no labor pains. The next day there was return of hemorrhage; she lost, as near as we could judge, ½ pint of blood. Os was now dilated, so that I could just introduce the point of my finger, and found placenta presenting. There were for 5 days slight discharges of blood, during which time I did not leave her for more than two hours at a time. The pulse was kept from rising above 70 by the occasional use of veratrum. On the morning of the 29th, about 8 o'clock, she had a severe pain; os dilating, (could feel the placenta covering the os,) and with the same pain a gush of blood and rupture of the membranes; placenta cleared itself from the left side of the os, the head of child pressed firmly down against the os; thus completely controlling the bleeding vessels. All this was with the first pain, and was the work of certainly not more than a minute. The labor went on naturally, and a few minutes before 10, A. M., she was delivered of a fine boy. The placenta was expelled naturally; and the uterus contracted firmly. I did not leave her for more than two hours, during which time she was cheerful and comfortable; had several after pains. Just before I left her I placed my hand on the abdomen, and found the uterus well contracted. In about an hour I was sent for, as she was thought to be flooding to death. I found her pale and pulseless; the abdomen as large as before delivery. Gave her immediately ʒj. of pulv. ergot, and raised the foot of the bed 18 inches above the floor, taking the pillows from under her head. The os was filled with a firm clot,—with one hand I turned this out, and with the other grasped the abdomen. There was a torrent of blood poured out, but the uterus did not contract. With my hand inserted into the vagina and three fingers in the os, I formed a tampon; cold water was poured on the abdomen; but no contraction took place. My friend Dr. Herd was in the next house, and I sent for him. He made firm pressure on the abdominal aorta, and with his hand and the use of ice, endeavored to make the uterus contract, which after a few minutes it did: but only to relax again. She now took another drachm of ergot. Several times in succession the uterus would contract and then relax for the space of near an hour, when it contracted firmly and permanently. There was no portion of placenta or membranes within the os; but it seemed as if a clot had formed there prior to the first relaxation of the uterus. I did not remove my hand from the vagina till the last firm contraction, and she was safe, though very low from the loss of blood. The point of interest in my own mind is,—what produced this peculiar condition of the uterus—was it from the veratrum which she had taken previous to her labor? If so, why did we not see its effects in arresting uterine contractions at first? The object I had in view in giving the veratrum was twofold—first from the pain in the head, and the rapidity of the pulse, I feared convulsions, as I had just passed through the case reported; and second, I thought by holding the circulation in check, to control somewhat the unavoidable hemorrhage when she came to labor.

---

*Puerperal Convulsions.—Post Partem.*

Case 2nd—Called at 5, P. M., Sept. 16th, to see Mrs. ——, a primipara. She had been delivered by a midwife at 10, A. M., the same day. I learned that before her delivery she had complained of severe head-ache and sense of heat in her head. Her husband at this time noticed that her pupils were much dilated, and that she had a wild, staring look, and called the attention of the midwife to it. She said "all was right;" but put her in a warm bath and bathed the top of her head with "Perry Davis' Pain Killer"—better call it "Woman Killer!" During the labor she was put under the influence of chloroform. I could not learn that there was anything peculiar about the labor. The pain in the head continued after labor, and about noon the midwife gave her a teaspoonful of the "pain killer" in a little water. In about five minutes she had a convulsion. As soon as she could swallow she was made to drink warm water. She soon vomited, and had another convulsion. When I arrived she had had five, and before I had time more than to look at her, she had another. I took from her arm 24 oz. of blood; the pulse softened down, and the comatose state did not last more than ten minutes. Gave her 15 grs. of calomel, and in an hour two table-spoonfuls of castor oil. She had no more paroxysms for three hours, when she had two in rapid succession. Bowels moved slightly; repeated the oil in two hours. She now had a convulsion. Opened the vein and took 20 oz. more of blood. She nearly fainted, and in a short time had two copious dejections. She had no more convulsions, but in less than three hours from the last she became furiously delirious, requiring to be held upon the bed. This was combatted by full doses of tr. veratrum viride and fluid extract valerian. In three hours she became calm, and slept for some hours. It was 48 hours before she became conscious even that she had been delivered, and did not recollect anything that transpired for three hours prior to the labor, though her attendants had supposed her to be perfectly rational. For several days there was much muscular soreness all over the body, and great tenderness of the abdomen, which was allayed by stupes of turpentine and laudanum. The urine was not tested for albumen.

In this case there was doubtless a strong tendency to convulsions, as evidenced by the severe and persistent headache. Had this been met at the outset by a free bleeding, there is little doubt the event would have been averted. As it was, through the ignorance and rashness of the midwife in bathing the head with, and exhibiting by the mouth, the powerful stimulant—it was hastened to a crisis. For two weeks prior to her confinement she had been daily subjected by the midwife to the use of an electro-galvanic machine, on the plea, that it would render her labor more easy. Her nervous system was strung up to the highest degree of tension, and she had been kept posted on the dangers of labor, and the untoward results which often took place. The arguments made use of to secure the valuable (?) services of the midwife, were, "that doctors were rash, and would be likely to use instruments," and that "it was very indecent for a young woman to have a man in attendance upon her."

---

### *Retroversion of Uterus.*

Case 3rd.—Called near midnight, Sept. 1st, to see Mrs. McC.——, Irish, mother of two children. Is now as she thinks in the tenth week of gestation. Two days previously, while hard at work, she "felt something wrong in her belly, and could not make water." Not knowing what was the matter, she commenced drinking herb tea, which she continued till the time of my visit. I found her screaming with pain—abdomen as large as at term—the bladder rising above umbilicus.

*Vaginal Examination.*—Found the vagina filled with a large tumor, and presenting beyond the vulva. Convinced that this was the retroverted womb, I tried first to introduce a catheter to relieve the bladder, but though the meatus was entered, the pressure on the urethra was so great that it was only by introducing my finger as high as possible, and endeavoring to press the womb back, that after long efforts I succeded, and brought away six quarts of water by measure, to her infinite relief. Though I could now insert my finger high up, I could not reach the cervex or os. Putting her in position, I introduced two fingers of the left hand into the rectum, and lifting the womb, while I made steady pressure with my right hand; after some time succeeded in raising the womb as high as the sacral promontory, and the vagina being very capacious, passed up my whole hand till the uterus was fully replaced. In a few minutes she had a fine movement of the bowels. Ordered her to keep her bed for a day or two, and left her. She soon felt as well as ever.

